# Smartphone app reveals that lynx avoid human recreationists on local scale, but not home range scale

**DOI:** 10.1038/s41598-022-08468-7

**Published:** 2022-03-21

**Authors:** Neri H. Thorsen, Richard Bischof, Jenny Mattisson, Tim R. Hofmeester, John D. C. Linnell, John Odden

**Affiliations:** 1 Norwegian Institute for Nature Research, Sognsveien 68, 0855 Oslo, Norway; 2 Faculty of Environmental Sciences and Natural Resource Management, Norwegian University of Life Sciences, P.O. Box 5003, 1432 Ås, Norway; 3 Norwegian Institute for Nature Research, Torgarden, PO Box 5685, 7485 Trondheim, Norway; 4 Department of Wildlife, Fish, and Environmental Studies, Swedish University of Agricultural Sciences, Skogsmarksgränd 7, 90736 Umeå, Sweden; 5 Department of Forestry and Wildlife Management, Inland Norway University of Applied Sciences, Anne Evenstads vei 80, 2480 Koppang, Norway

**Keywords:** Ecology, Behavioural ecology, Conservation biology

## Abstract

Outdoor recreation is increasing and affects habitat use and selection by wildlife. These effects are challenging to study, especially for elusive species with large spatial requirements, as it is hard to obtain reliable proxies of recreational intensity over extensive areas. Commonly used proxies, such as the density of, or distance to, hiking paths, ignore outdoor recreation occurring on other linear feature types. Here we utilized crowdsourced data from the Strava training app to obtain a large-scale proxy for pedestrian outdoor recreation intensity in southeast Norway. We used the proxy and GPS-tracking data from collared Eurasian lynx (*Lynx lynx*) to investigate how recreation affects habitat selection at the home range scale and local scale by lynx during summer. We fitted resource selection functions at the two scales using conditional logistic regression. Our analysis revealed that lynx avoided areas of recreational activity at the local scale, but not at home range scale. Nonetheless, lynx frequently used areas associated with recreation, and to a greater degree at night than during the day. Our results suggest that local-scale avoidance of recreation and temporal adjustments of habitat use by lynx mitigate the need for a home range-scale response towards recreation. Scale-dependent responses and temporal adjustments in habitat use may facilitate coexistence between humans and large carnivores.

## Introduction

Large carnivores living in human-dominated landscapes face widespread land-use changes, infrastructure, and the presence of humans. The ability of large carnivores to share the landscape with humans will be increasingly challenged, as the human population is growing^1^. Land-use changes and human infrastructure impact the behaviour, habitat use, and habitat selection of large carnivores^2^. Although less conspicuous, outdoor recreation constitutes another disturbance pressure on wildlife in the human-dominated landscape. Despite being more benign than, for example, hunting and habitat conversion, recreation also affects behaviour, habitat use, and habitat selection^3^. For some wildlife species the negative impact from recreation is even considered an important conservation issue^4^ (e.g. Barbary Macaque *Macaca sylvanus*^5 ^and cheetah *Acinonyx jubatus*^5^). Hence, understanding the effects of recreation on wildlife is crucial for mitigating its potential negative impacts at present and in the future.

The vast extent and intensity of outdoor recreation is illustrated by an estimate of 8 billion visits solely to protected areas per year globally and 4 billion visits per year in Europe alone^6^. Outdoor recreation is rising^7^ and, in combination with increasing urbanisation^8,9^, the patterns will likely change in the future, with growing levels of outdoor recreation especially around urban areas. These changes will have implications for wildlife. The effects of recreation on wildlife have received increased research attention throughout recent decades^10^ and a wide range of effects of recreation on wildlife has been documented^3^. Generally, animals tend to respond to humans in the same way they respond to a predator^11^. The detection of a human is usually followed by a behavioural and/or physiological response^12,13^. These responses might in turn influence energy budgets, habitat use and fitness. Hence, recreation can in the worst case influence population trends and the distribution of animals^3^.

Habitat selection, i.e. the disproportional use of a habitat type relative to its availability, can be inferred at multiple scales based on how availability is defined^14^. Animals may respond to the same habitat feature differently based on the scale of habitat selection^15^. For instance animals may avoid large patches of habitat that contain many linear features or receive high levels of recreation at the home range-scale^16^ (cf. 3rd order of habitat selection^14^). Animals may also avoid specific linear features that are associated with high levels of recreation at a local scale^17,18^ (cf. 4th order of habitat selection^14^), even if not avoided at a larger scale. To understand at which scale animals respond to recreation is therefore important for coexistence of humans and wildlife, as the ability to respond to recreation at the local scale^19^ may mitigate disturbance effects at the home range scale.

Large carnivores are now recovering in Europe from past persecution and returning to a human-dominated landscape^20^. Despite large carnivores being apex predators, humans influence their behaviour^21^ and ecosystem function^22^. Numerous studies have shown behavioural and physiological responses in large carnivores when humans directly approach on foot (e.g. brown bears *Ursus arctos*^13,23^, black bears *Ursus americanus*^24^, wolf *Canis lupus*^25^ and puma *Puma concolor*^26^), but less attention has been paid to how habitat selection and habitat use by large carnivores are affected by human recreational activity and not only the infrastructure associated with recreation. Recreation can reduce habitat quality^27^ and spatial avoidance of suitable habitat due to recreation can be functionally equivalent to habitat loss^16,28^. Large-scale segregation due to recreation has been documented for group-living herbivores in open landscapes^29^ and for wolverines (*Gulo gulo*) in mountain areas^16^ due to functional habitat loss. However, it is unclear how large carnivores, with large space requirements, respond to outdoor recreation over different scales in forested habitats.

The paucity of studies on the effect of recreation on habitat selection by large carnivores thus far is likely in part due to the lack of measures of human activity or recreation for the large areas in which these species live. Path density or distance to paths have been commonly used as proxies for human recreation^10,30^. These proxies ignore the fact that different path segments are associated with different levels of recreation and that other linear features than paths are also associated with recreation, such as forest roads and public roads. Estimating the level of recreation along linear features with, for example, human counters^31^ or camera traps requires vast resources if it is to be done at a scale relevant for large carnivores. Today, ubiquitous smartphones and smartwatches with built-in GPS-loggers have opened up new possibilities to obtain proxies for human activity at large spatial scales^32^. Users of certain software applications (apps), like the training app Strava (www.strava.com), agree to share their spatial locations with the company. For apps with large userbases, such data can provide relative proxies for the spatial distribution of recreational activity^33,34^. Here we utilize data from the Strava app as a proxy for pedestrian outdoor activity (walking, running, or hiking) during summer in southeast Norway and investigate how habitat selection and habitat use by Eurasian lynx (*Lynx lynx*) are influenced by recreation. Furthermore, we included density of hiking paths as a proxy for recreation and tested whether lynx responded differently to these two proxies of human recreation.

The Scandinavian lynx population has recovered after being hunted to the edge of extirpation in the mid-twentieth century^35^. The population continues to be exposed to extensive legal hunting, and human-caused sources of mortality are high (poaching and vehicle collisions in addition to hunting)^36^. Hence, there is a potential for strong avoidance of humans in this population. We studied the effect of summer recreation on habitat selection by lynx with resource selection functions (RSF^37^) at two spatial scales with different availability definitions; within the home range (home range-scale) and within a buffer of 1–2 km around locations used by the lynx (local-scale). If the lynx manage to coexist with humans by locally avoiding features with high recreational activity, they can still occupy human-dominated landscapes, which will facilitate coexistence compared to a total avoidance of humans. Furthermore, we investigated whether habitat selection was influenced by the time of day. Lastly, we explored how lynx habitat use of areas associated with recreation changed throughout the day.

## Methods

### Study area

**Figure 1:**
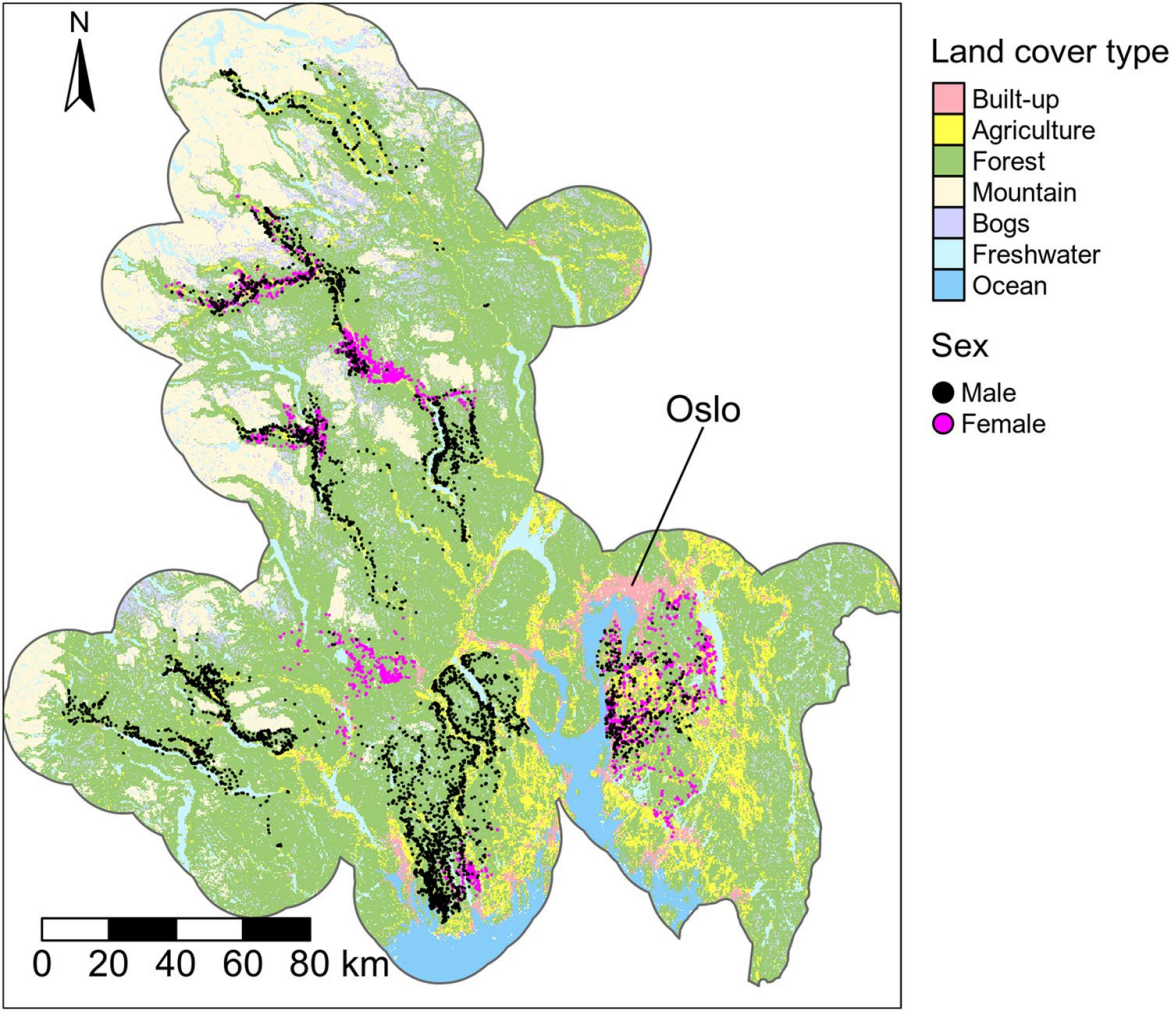
Location of the study area (coloured region) and GPS-locations of lynx (black and pink dots). The study area was delineated by drawing an 18-km buffer around all lynx GPS-locations. The figure was created using the R package tmap (version 3.2, https://cran.r-project.org/web/packages/tmap/index.html).

The study area (approximately 43,000 km^2^) is located in southeast Norway (centroid coordinate: N 59.96982, E 9.693853), in Innlandet, Vestfold og Telemark, Oslo, and Viken counties (Fig. 1). The study area includes the most heavily populated areas in Norway, and has an overall human population of 2 million (www.ssb.no). In the southeast part of the study area, the landscape consists of forest fragmented with agricultural land and settlements, and a rolling topography. In the northwest, the topography is characterized by steep slopes, valley systems and some agricultural land along the valley floors. Most of the forests are heavily exploited by commercial forest industry and associated clearcut practices. The main tree species are Norway spruce (*Picea abies*), Scots pine (*Pinus sylvestris*) and birch (*Betula* sp.). Roe deer (*Capreolus capreolus*), free-ranging domestic sheep (*Ovis aries*), red deer (*Cervus elaphus*), and small prey species such as mountain hare (*Lepus timidus*), tetraonids and other birds comprise the diet of the lynx in the area^38,39^.

### Animal capture and GPS-data

From 2008 to 2014, 25 lynx (11 females and 14 males) were captured in foot snares or wooden box traps and equipped with GPS-collars following a pre-established protocol^40^. All capture and handling procedures were approved by the Norwegian Experimental Animal Ethics Committee (permit numbers 2012/206992, 2010/161554, 2010/161563, 08/127430, 07/81885, 07/7883). This study was conducted in compliance with the ARRIVE guidelines, and all our methods were performed in accordance with relevant guidelines and regulations. In this study, we only analysed data from adult lynx in years when they were settled in a home range. GPS-collars were programmed to take between 1–19 GPS-locations per day. Fix schedules varied across individuals and alternated between intensive predation study periods and less intensive monitoring periods^39^. As the aim of this study was to explore how recreation affects habitat selection by resident lynx during the snow-free period, we included observations from 1^st^ May to 31^st^ October during all all years. To increase the robustness of the analyses, we only included combinations of lynx individuals and years (i.e. lynx-years) with ≥ 200 GPS-locations. This resulted in a final dataset of 13 611 GPS-locations for 22 lynx-years from 20 individual lynx (8 females and 12 males). Two females were included during two years each.

### Recreation

**Figure 2:**
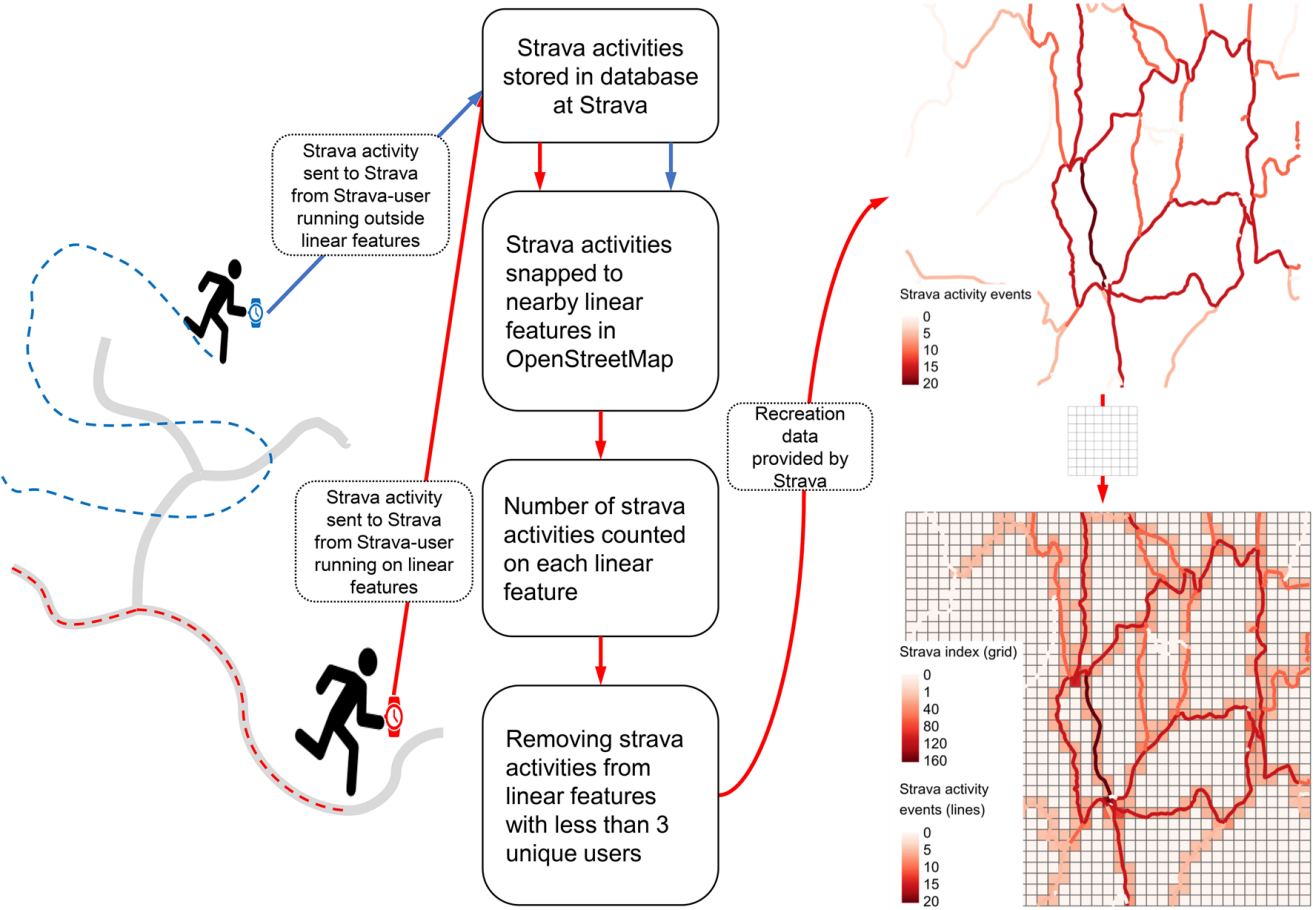
Schematic presentation of the recreation data provided by Strava. The grey linear features to the left represent the linear features in the OpenStreetMap (OSM). The dotted lines represent the track left by two Strava-users, where the blue is from a user running outside linear features and the red is from a user running on linear features. The activity event from the blue user cannot be snapped to linear features in OSM and is removed during processing. The map to the top right represents a network of linear features and associated Strava activity events. The legend shows how many activity events are associated with the different paths (note that they are rounded to nearest multiple of 5). The map in the bottom right represents the same network after the number of Strava activity events for every linear feature in each grid cell have been counted.

We used crowdsourced human mobility data from Strava as a proxy for recreation. Strava is an app for smartphones and smartwatches, used primarily to record and upload georeferenced human training activities (hereafter referred to as “activity event”). Activity events can also be uploaded directly to the Strava webpage. Strava stores this data and processed version of it and can be accessed from Strava Metro. To maintain anonymity and conform with privacy regulations, access is limited to data processed by Strava through removal of personal identifiers and spatial and/temporal aggregation (Fig. 2). The processing involves linking individual activity events to nearby linear features (paths, roads etc.) in OpenStreetMap (OSM, www.openstreetmap.org). Hence, in the absence of OSM linear features close to an activity event (or parts of it), that event (or parts of it) is not included in the aggregated version of the dataset (Fig. 2). Further, to conform with privacy legislations, linear features with less than three unique users are removed and the number of activity events are rounded up to the nearest multiple of five. Based on our initial inspections of the Strava data, pedestrian activity (walking, running, hiking etc.) appeared to extend further into the forest compared to biking. Hence, we decided to focus our analysis only on pedestrian activities. We used Strava activities from pedestrians at a temporal resolution of one year, i.e., data where the timespan for at least three unique Strava users on a linear feature was a year, to maximise the spatial coverage. We included Strava data from 2016 to 2019 and summed this over all years to result in a single static covariate. A more detailed description of how we processed the Strava data can be found in Supplementary material S1 and Figure S1.

We also used the density of hiking paths as a proxy for recreation to facilitate comparison of a more traditional method with the Strava data. We obtained data on hiking paths from the OSM (key = highway and value = track and path) using the *osmdata* package^41^ in R (version 4.0.3^42^). To derive our proxies for recreation, we divided our study area into a grid of 50 × 50 m (a compromise between computational time and highest possible resolution). For hiking paths, we calculated the line lengths of hiking paths inside each grid cell. For the Strava data, we summed up all the pedestrian activity inside each grid cell. We refer to this covariate as the Strava index. The value of this index in a given grid cell can be viewed as the total number of activity events taking place on all linear features within that grid cell during the entire period when the Strava data were collected. The Strava index accounts for different levels of human activity associated with all types of linear features (not only hiking paths) inside a grid cell, while the hiking path density is only a measure of the length of the hiking path inside a grid cell and does not account for the level of associated human activity.

### Resource selection functions at two scales

**Figure 3:**
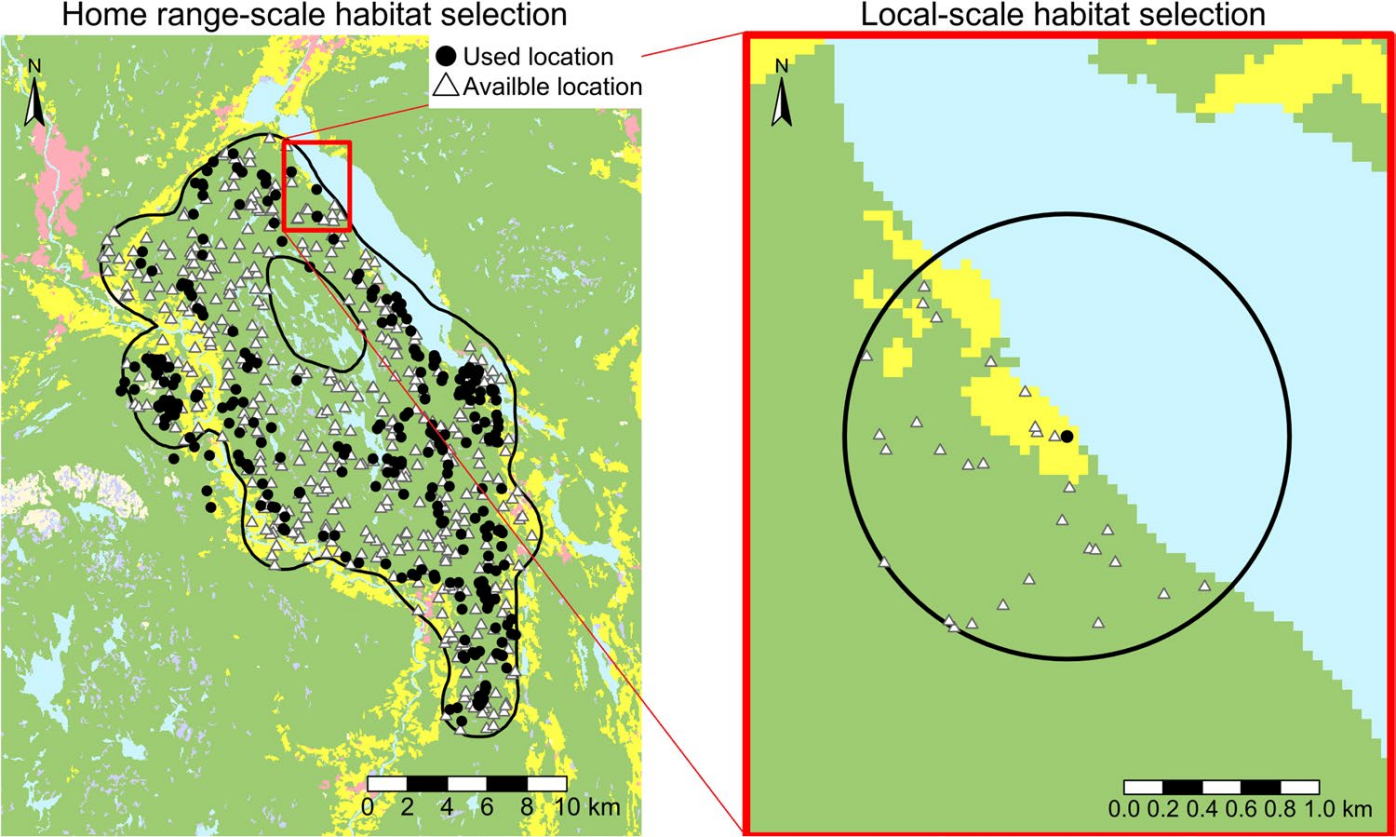
Illustration of the different scales and availability definitions. For the home range-scale analysis (left map) we sampled 30 available locations per used location inside the home range of the lynx (for illustration purposes we used a ratio of 1:1 in the left map). While for the local-scale analysis (right map) we sampled 30 available locations inside a buffer with the radius equal to the mean step length (distance between two consecutive locations) of the lynx (for illustration purposes we removed two buffers and their corresponding points in the right map). The figure was created using the R package tmap (version 3.2, https://cran.r-project.org/web/packages/tmap/index.html).

We used resource selection functions (RSF^37^) to estimate habitat selection by lynx. RSF depends upon a use-available design, where locations used by GPS-collared lynx are compared to locations not used but considered as available (“null model”). For each used GPS-location, we randomly sampled 30 available locations (excluding open water). Based on our sensitivity analysis^43^ the choice of 30 available locations per used location appeared to be sufficient (see Figure S2–S3). We had two different availability definitions (Fig. 3). For the home range-scale habitat selection we sampled available locations inside the home range. Home ranges were estimated with the Brownian bridge kernel method^44^ with the *kernelbb* function in the *adehabitathr* package^45^ in R. We used the 95% isopleths. For the local-scale we sampled available locations inside a buffer with the mean step lengths (distance between two consecutive GPS-locations) of the individual as radius from the used GPS-location (Table S1). We then fitted conditional logistic regression to obtain the RSFs by using the *coxph* function in the *survival* package^46^ in R. The response was whether a location was used (1) or available (0). Conditional logistic regression relies on a matched design, where groups of observations are matched with given grouping IDs. For the home range-scale we matched used and available locations by including the lynx-year ID as strata (not to be confused with the Strava index) and for the local-scale we used an unique identifier for the buffer as the strata. This ensured that used locations in a home range were only compared to available locations sampled from the same home range, and also that the used location in a buffer was only compared to the available locations in the same buffer. Furthermore, we included the lynx-year ID as a cluster variable in the models to obtain robust standard error estimates for the coefficients^47,48^. We interpreted robust confidence intervals overlapping 0 as a lack of evidence for either avoidance or selection.

### Covariates

Based on findings previously reported for lynx^30,49–51^, we considered the following covariates: slope, agricultural land (hereafter referred to as fields), forest, forest roads, public roads, and houses. In addition, we also considered the Strava index and hiking path density as proxies for recreation. Spatial data on fields and forest were obtained from an open access land cover map (AR50^52^, 1:50,000); and vector data on public roads, forest roads and houses from the Norwegian Mapping Authority (www.geonorge.no). We defined a house as a building approved as a residential building for the entire year. This excludes recreational cabins, which are associated with a variable degree of human activity. Slope was calculated with the *terrain* function (based on the 8 nearest neighbouring raster cells) in the *raster* package in R^53^, based on a digital elevation model with 50 m resolution from the Norwegian Mapping Authority (www.geonorge.no). All covariates were rasterized to a 50 × 50 m resolution (see Table S2 for simple summary of the covariates).

We prepared the covariates differently for the home range- and local-scale (see Table S2), to account for the different definitions of available habitat. For the home range-scale analysis we calculated the density of houses, forest roads and public roads, the proportion of forest, and the proportion of fields in a buffer of 1 km radius around each cell (50 × 50 m). For hiking path density we calculated the sum of all cells in a buffer of 1 km radius around the focal cell, while for the Strava index and slope we used the mean. We denote the hiking path density, Strava index, and slope with the subscript 1000 (e.g., Strava index_1000_) to separate them from the covariates with similar names used for the local-scale. For the local-scale analysis we used distance to house, distance to forest road, distance to public road and distance to field, instead of density or proportion metrics. We included forest as a binary variable (1 = forest and 0 = not forest). For the Strava index and hiking path density we calculated the mean (Strava index) or sum (hiking path) of the covariate in the four closest neighbouring cells and the focal cell. This ensured that the hiking path density and Strava index extended at least 50 m outside the linear feature they were associated with. For slope on the local-scale we used the original calculation as previously described. We denote the Strava index, hiking path density and slope used for the local-scale analysis with the subscript 50 (e.g., Strava index_50_) to separate them from the covariates used in the home range-scale analysis. In addition, we also considered day vs. night as a covariate in models at the local-scale. Night was defined as the time between sunset and sunrise, obtained by the sunriset function in R-package maptools^54^. We standardized all the continuous covariates by subtracting the mean and dividing by the standard deviation of all used and available locations prior to fitting the models. The distance to feature covariates were log-transformed, to make the effect of the covariate decrease with the distance from the feature (we added 1 m to all distances prior to log-transformation).

### Candidate models and model selection

We considered four different candidate models for the home range-scale and eight different models for the local-scale analysis (see Table 1). The simplest model (“core model”) contained parameters that have previously been shown to be important for habitat selection by lynx. For both scales, we tested whether including recreation covariates improved our core model by adding the density of hiking paths (“path model”), the Strava index (“Strava model”) or both the density of hiking paths and the Strava index (“full model”). In the core model for the home range-scale we included the quadratic term of the proportion of forest as the results of Bouyer et al.^51^ suggested selection for an optimum less than 100% forest cover. In addition, we tested if local-scale habitat selection was influenced by time of day by including an interaction with night (true/false) for slope and forest as Filla, et al.^30^ showed that lynx select gentler slopes and spend more time in open habitats during the night. Given the diurnal activity of humans, we also included an interaction with night for the following human related covariates: distance to public and forest roads, hiking path density_50_, and the Strava index_50_. We did not include a main effect of night in the model as this variable was constant for any given stratum (single used location and associated sample of available locations) and could therefore not be meaningfully evaluated. We used Akaike’s Information Criterion (AIC^55^) to rank models with the strongest support, and based inferences on models ≤ 2 delta AIC of the top ranked model^56^. We interpreted that including recreational activity improved our models when either the full-, Strava-, or path model ranked highest or ≤ 2 delta AIC off the top-ranked model.

**Table 1:** Candidate models for home range-scale and local-scale habitat selection

Model	Model specification home range-scale	Model specification local-scale
Core	Slope_1000_ + forest cover + (forest cover)^2^ + field cover + forest road density + public road density + house density	Slope_50_ + forest (dummy variable) + distance to fields + distance to house + distance to forest road + distance to public road
Path	Core + hiking path density_1000_	Core + hiking path density_50_
Strava	Core + Strava index_1000_	Core + Strava index_50_
Full	Core + hiking path density_1000_ + Strava index_1000_	Core + hiking path density_50_ + Strava index_50_
Core_ night		Distance to fields + distance to house + slope_50_ : night + forest : night + distance to forest road : night + distance to public roads : night
Path_night		Core_night + hiking path density_50_ : night
Strava_night		Core_night + Strava index_50_: night
Full_night		Core_night + hiking path density_50_ : night Strava index_50_ : night

Refer to Table S2 and S3 to see how the covariates were prepared for the different scales. The night interaction was only considered at the local-scale.

### Time dependent habitat use of areas associated with Strava use

In addition to habitat selection, we explored how the habitat use of areas associated with the Strava index_50_ changed throughout the day. This was done by fitting a generalized additive mixed model (GAMM^57^) to only the GPS locations used by the lynx. As response, we used the GPS-locations of the lynx and coded them as 1 if the location had Strava activity (Strava index_50_ > 0) and 0 if not. We used a binomial distribution with a logit link to model the response. The predictors included hour of the day with a cyclic spline as a smoothing term and lynx ID as a random intercept. We used the *gamm* function in *mgcv* package^58^ in R to fit the model. The time of day was corrected for differences in day length using two anchors (one at sunrise and one at sunset) and the average method^59^ in the *activity* package^60^ in R.

## Results

Mean human density within lynx home ranges was 26 km^-2^ (range: 4.8–166 km^-2^). On average, 12% (range: 0.8–31%) of the used GPS-locations per lynx, and 16% (range: 5.7–37%) of lynx home range areas, were located in grid cells with Strava index_50_ > 0. Corresponding values for Strava index_1000_ > 0 were 82% (range: 40–99.5%) of the used GPS-locations per lynx and 79% (46–98%) of their home range area.

Of the Strava activity events inside the lynx home ranges, 58% were located in forest, 24% occurred in built-up areas (urban, sub-urban, small towns etc.), 11% on fields, and 5% in alpine areas (open areas above the forest). Inside the lynx home ranges, roads were the linear features that had the highest levels of activity events, with hiking paths receiving fewer activity events (Figure S4).

### Home range-scale habitat selection

The four candidate models differed only moderately from each other in terms of AIC (Table S4). The model including hiking paths ranked highest, but the full model was within 2 ΔAIC. We therefore present the results from the full model. In addition, the direction of effects (when included) was similar in both models.

**Figure 4:**
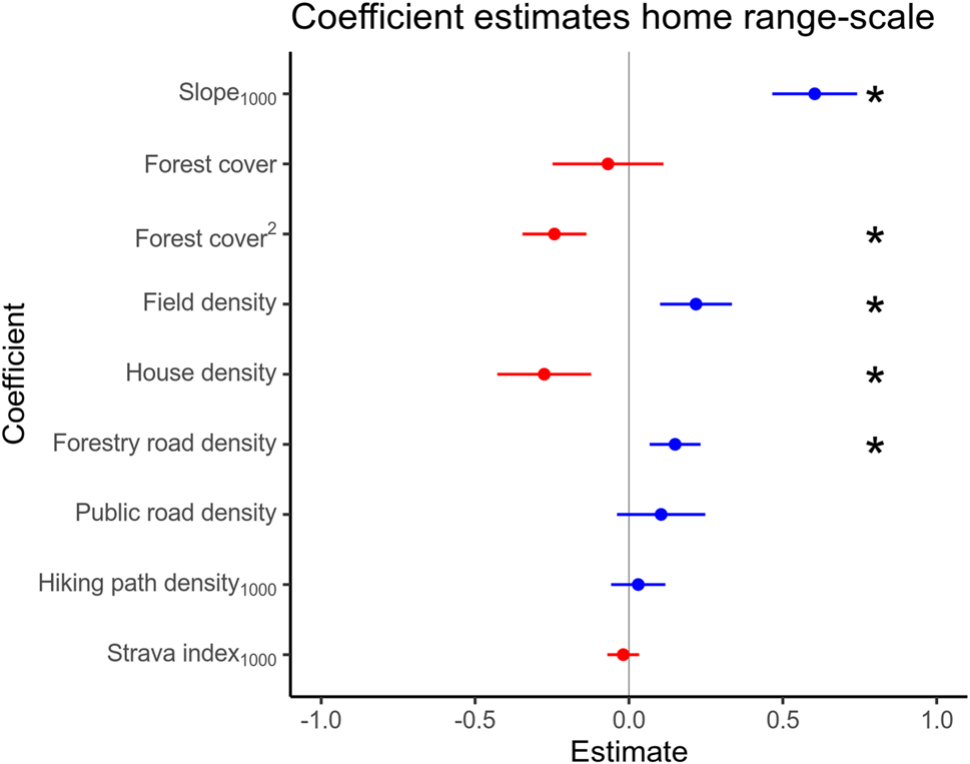
Selection coefficients for home range-scale habitat selection. Point estimates and 95% confidence intervals based on the robust standard errors for the home range-scale RSF models. Red colours indicate negative value of the estimate (i.e. avoidance) and blue colours indicate positive values of the estimate (i.e. selection). Stars indicate significant estimates at the alpha level of 0.05.

We did not detect evidence that hiking path density_1000_ or Strava index_1000_ significantly influenced habitat selection at the home range-scale (Fig. 4, *P* value 0.502 and 0.483, respectively). Lynx selection increased with steeper slope (slope_1000_, *P* value < 2*10^–16^), higher proportion of fields (*P* value 2.56*10^–4^), higher forest road density (*P* value 3.74*10^–4^), lower house density (*P* value 3.96*10^–4^) and they selected forest cover with an optimum around 77% forest (see Fig. 6A, *P* value 0.458 and 4.86*10^–6^ for first and second order term, respectively). We did not find evidence that public road density significantly influenced habitat selection by lynx at the home range-scale (*P* value 0.153).

### Local-scale habitat selection

**Figure 5:**
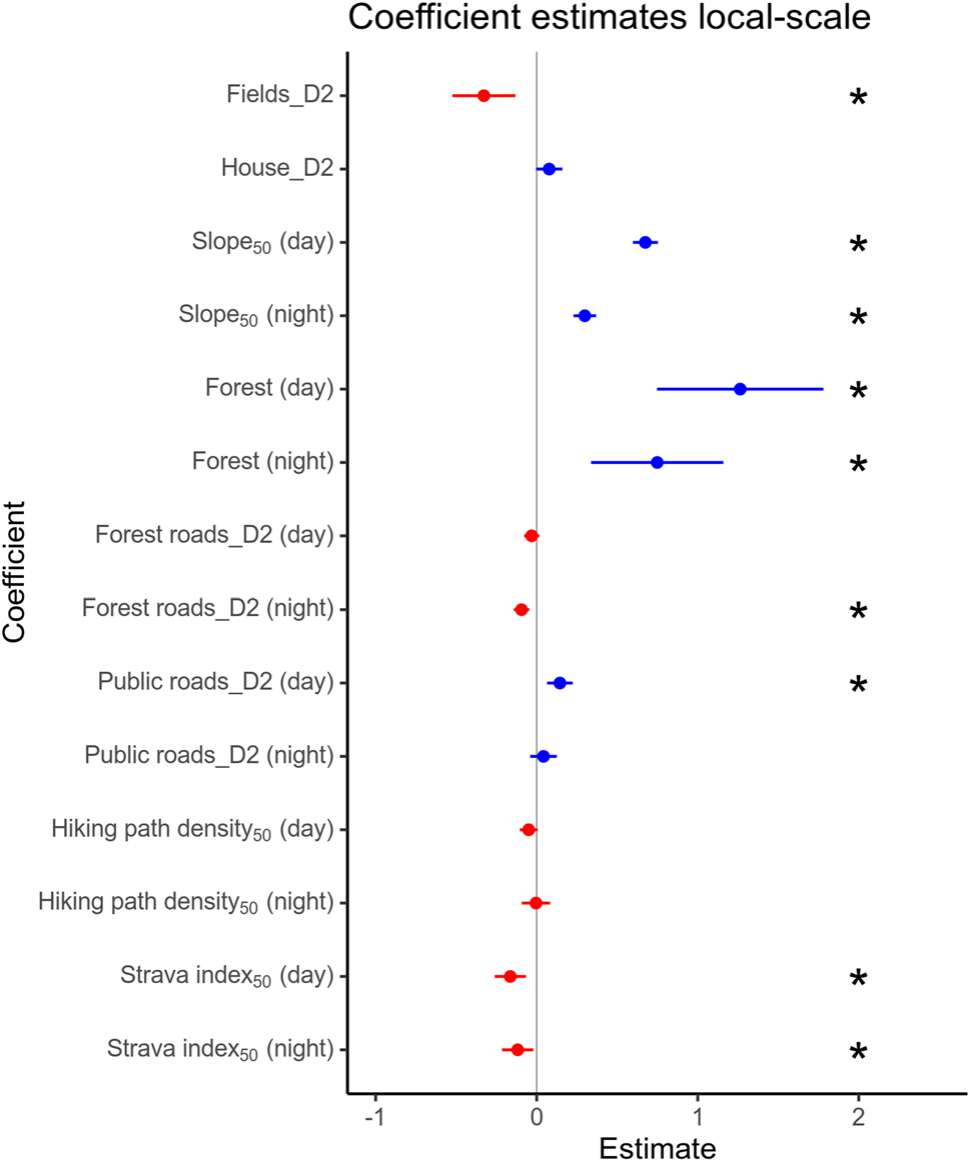
Selection coefficients for local-scale habitat selection. The point estimates and 95% confidence intervals based on the robust standard errors for the local-scale RSF models. Red colours indicate negative value of the estimate and blue colours indicate positive values of the estimate. D2 is an abbreviation for “distance to”, all “distance to” features have been log-transformed to make the effect decrease with large distances. Note that a positive estimate for distance to feature indicates avoidance (selecting for areas farther away for the feature). Stars indicate significant estimates at the alpha level of 0.05.

The full model including interaction with time of day emerged as the top model for local-scale habitat selection (Table S4) and was clearly the most supported model. Models including “night” and/or the Strava index performed better than those without. Lynx avoidance increased with higher Strava index_50_ during both day (*P* value 9.51*10^–4^) and night (*P* value 0.017, Fig. 5). The effect of hiking path density_50_ was not significant (P value 0.079 and 0.920 for day and night, respectively), regardless of the time of day, although there was a trend towards avoidance during the day.

**Figure 6:**
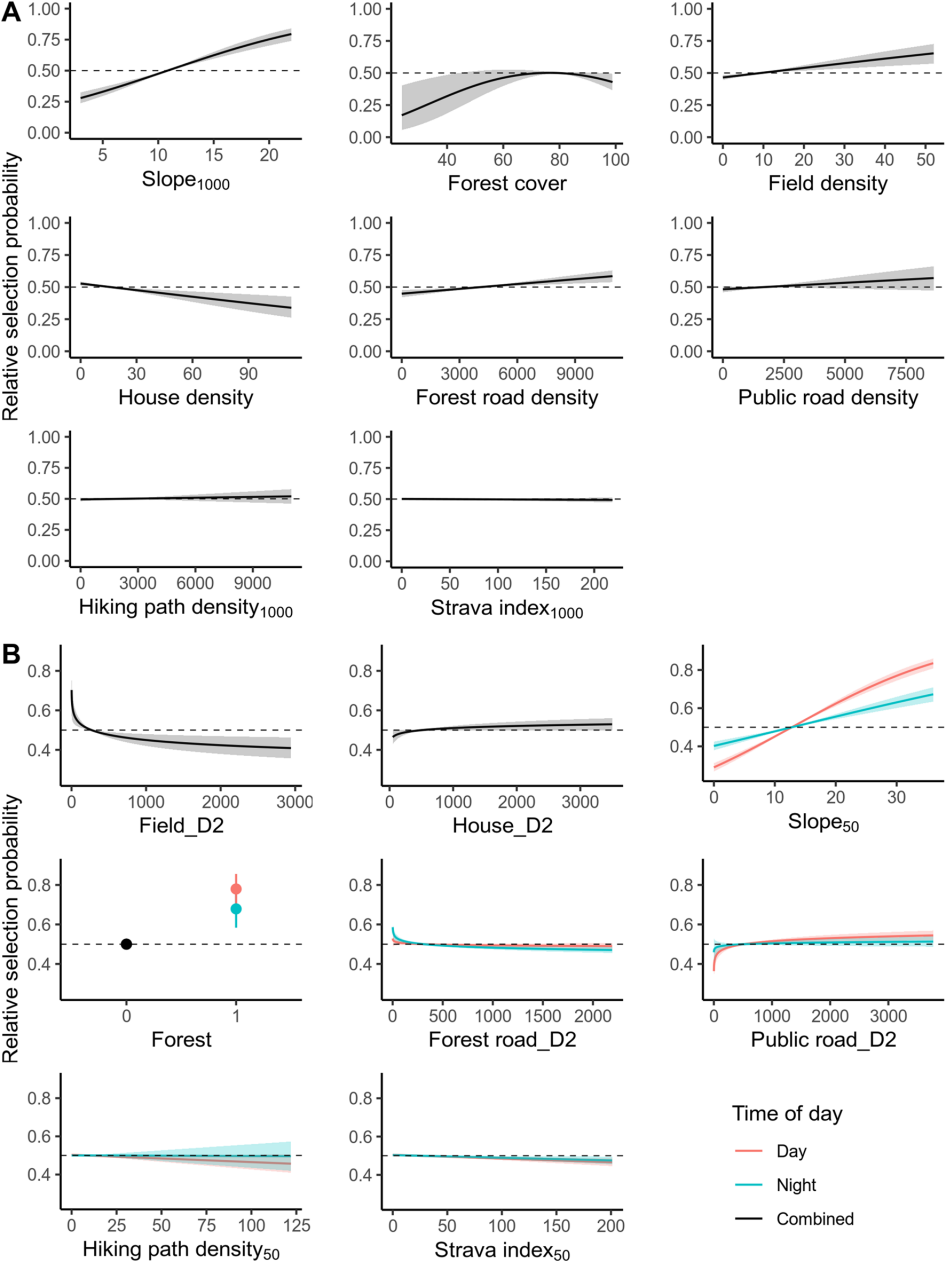
Predicted relative selection probability for each covariate for the home range-scale (**A**) and local-scale (**B**) habitat selection and their 95 % confidence interval. The range of the covariates (x-axes) is their 2.5 and 97.5% percentiles. Probabilities above the dashed horizontal line indicate selection and probabilities below indicate avoidance, relative to the “reference cell”. The reference cell, which all probabilities are relative to, is a cell where all the covariates are at their mean (due to the standardization prior to model fitting), and is not the same for home range-scale and local-scale habitat selection.

Lynx selection decreased with distance to fields (Fig. 6B *P* value 0.001). We did not detect an effect of distance to house on local-scale habitat selection (*P* value 0.063). Lynx selection increased with steeper slope (*P* value day < 2*10^–16^ and night < 2*10^–16^) and in forest (*P* value day: 1.63*10^–6^ and night: 3.61*10^–4^), but this effect was weaker during the night. During the day, lynx selection increased with distance from public roads (avoidance, *P* value 3.95*10^–4^) but showed no response to forest roads (*P* value 0.195), whereas at night, lynx selection decreased with distance to forest roads (selection for, *P* value 1.17*10^–4^) but showed no response to public roads (*P* value 0.318).

### Time dependent habitat use of areas associated with the Strava index_50_

**Figure 7:**
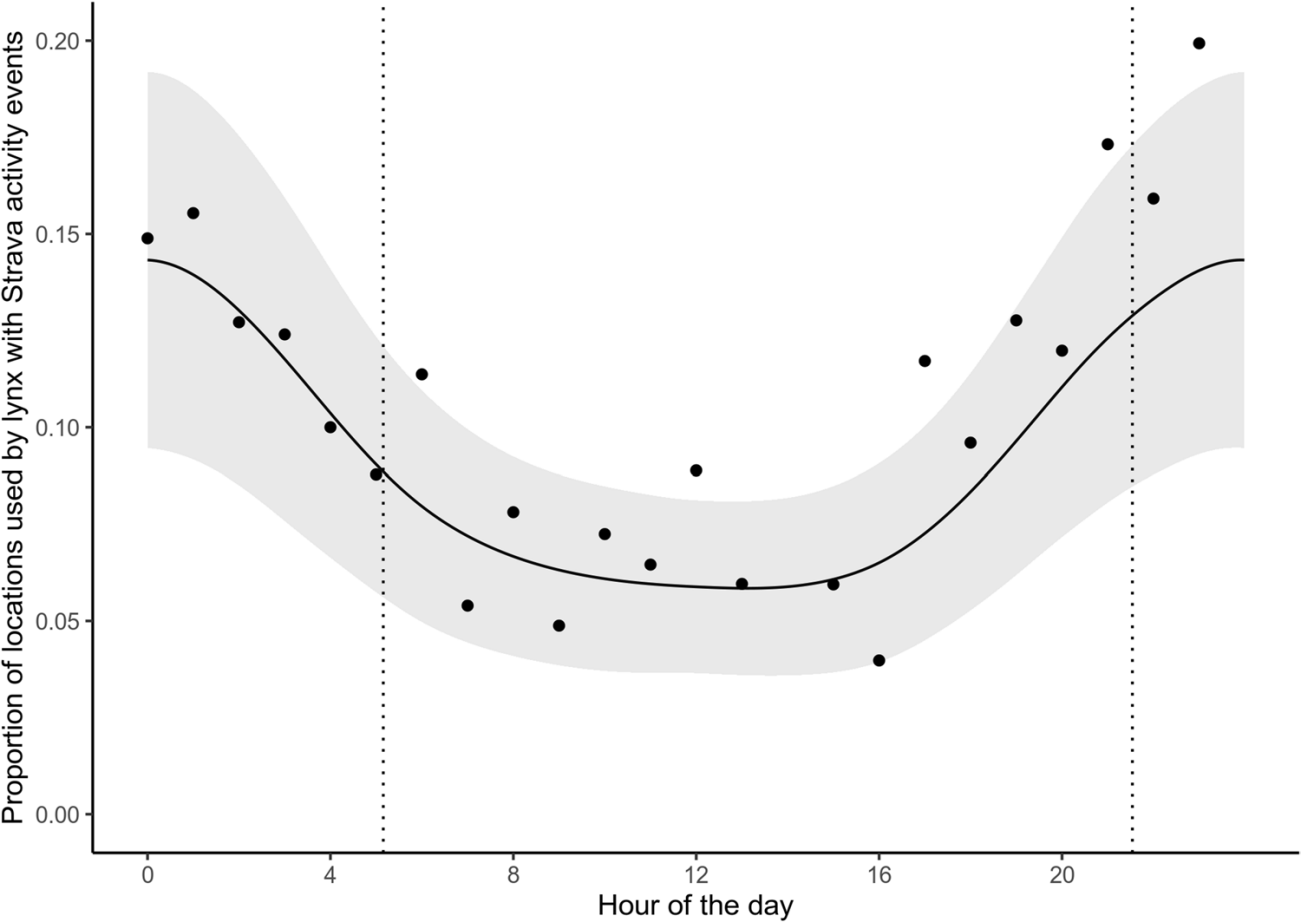
Prediction (black line) from the generalized additive mixed model (GAMM) with GPS-locations as a response, coded 1 if they were located in a grid cell with Strava index_50_ > 0 and 0 if not. The y-axis shows the proportion of locations used by lynx that were associated with recreation. Hour of the day was included as an explanatory variable and lynxID as a random effect on the intercept. The shaded area represent the 95% confidence interval around the prediction. Black dots indicate the proportion of GPS-locations in grid cells with Strava index_50_ > 0 during a given hour; only hours with more than 100 GPS-locations are shown. All times have been corrected for differences in day lengths with two anchors (sunrise and sunset). Sunrise and sunset are shown as vertical dotted lines.

Despite lynx selection decreasing with the Strava index_50_ at the local-scale both during day and night, lynx still used areas with a Strava index_50_ > 0. The GAMM revealed a time-dependent use of these areas (Fig. 7). The proportion of lynx locations having a Strava index_50_ higher than 0 was lowest during the day, from around 08:00 to 16:00 when predicted proportion was in the range of 0.06 to 0.07. From 16:00 to 00:00 the proportion increased and reached a peak of 0.14 around 01:00, after the peak the proportion declined until 08:00.

## Discussion

Our study revealed that lynx exhibit local-scale avoidance of areas with high levels of recreation in summer. Interestingly, while this effect was pronounced at the local-scale habitat selection, we did not detect any effect of recreation on home range-scale habitat selection. These results suggest that lynx are capable of adjusting their habitat selection and temporally adjusting their habitat use to recreation in a way that allows them to occupy human-dominated landscapes. This study also illustrates the added value presented by crowdsourced human mobility data as a reliable proxy for human activity in ecological studies, and the importance of accounting for the level of recreation associated with linear features of all types.

Accounting for the intensity of recreational use is important when studying the impact of recreation on wildlife, as the level of recreation can affect the animals’ responses^17^. The high level of recreational activity observed on other linear features than hiking paths suggests that using hiking paths as proxies for summer recreation fails to cover the entire spectrum of recreation. The inclusion of the crowdsourced human mobility data in our study revealed new details about the habitat selection and habitat use of lynx, similar to a recent study on brown bears (*Ursus arctos*) in Italy^33^. Lynx appeared to be relatively tolerant towards recreation, as we detected comparatively high use of the areas associated with the Strava index_50_ (11% of the used locations had values higher than 0). Due to the lack of avoidance at the home range-scale, lynx do not seem reluctant to occupy the same areas that humans use for recreation, but they do avoid the immediate surroundings of linear features associated with high levels of recreation (local-scale avoidance).

Previous studies have investigated the effect of recreation on home range-scale habitat selection and habitat use of large carnivores. These studies have reported selection for areas with nonmotorized winter recreation by Canada lynx (*Lynx canadensis*)^61,62^, avoidance of areas with higher intensity of winter recreation (both motorized and non-motorized) by wolverines^16^, avoidance of areas with higher recreational intensity by brown bears^33^ and daybed selection for areas assumed to receive less recreational activity by lynx^63^. In this study, we did not detect any spatial avoidance of hiking path density nor Strava index_1000_ (in a 1 km buffer) by lynx at the home range-scale habitat selection. Lynx have been reported to have relatively short flight initiation distances in forests, with a median distance at 50 m^64^. Hence, a substantial reduction in the need to initiate a flight response is likely achieved by local-scale avoidance of areas with high recreation levels at local-scale habitat selection. This local-scale avoidance might mitigate the need to exhibit larger scale avoidance; instead of avoiding a large forest area associated with high levels of recreation, lynx can still use it and can reduce the risk of encountering humans by local-scale avoidance of recreationists and temporal adjustments of habitat use.

During the day, local-scale avoidance of recreation likely reflects a selection of resting sites away from areas with high recreational use, which has also been reported for lynx in southern-Europe^63^. We found that lynx avoidance of areas with higher Strava index_50_ persisted throughout the night, at times when lynx are most active^65^ and humans are not. A lack of temporal adjustment in habitat selection towards areas associated with recreation (non-motorized and motorized recreation during winter) has also been reported for Canada lynx^61^. However, our results show that lynx, despite locally avoiding areas associated with higher levels of recreation, used areas associated with recreation quite often. Especially at night, when habitat use of Strava index_50_ was twice as high than during the day, showing some temporal adaptations towards recreation. Higher habitat use of areas associated with the Strava index_50_ could be explained by cost-effective transportation, as linear features have been shown to facilitate movement for other carnivores^15,66^.

Lynx in our study area occupy a human-dominated landscape and are thus capable of adjusting to human infrastructure^67^ and, as our results suggest, also recreation. The level of recreation in our study area might not be high enough to force lynx to adjust their habitat selection at larger scales. Nonetheless, our study area contains one of the most heavily used recreational areas in the immediate proximity of the capital of Norway (Oslo), and lynx still used this area. Additionally, the Scandinavian lynx population has been, and still is, subject to strong selection pressure to avoid humans due to hunting and poaching^35,36^. In this context, a lack of avoidance at the home range-scale habitat selection suggests that an area needs to receive substantially high levels of recreation before lynx start to avoid it at large scale, and that the other spatial and behavioural adaptations are sufficient. Our study area is forested, with abundant hiding cover and widespread access to rugged terrain and/or boulders. Dense horizontal cover has been shown to reduce the flight initiation distance for lynx^64^ and other large carnivores^12^. Hence, the effect of recreation might be less pronounced in forested landscapes with access to cover.

Strava data have proven useful in previous studies, and high correlations with ground truth data have been reported from cities in Norway^68^, the UK^69^, the USA^70^ and Australia^71^ as well as in rural areas in Austria^34^ and in Italy^33^. As our Strava index is an index of pedestrian recreation and the app is not used by everyone engaging in recreational activities in a defined area, true recreational activity is bound to be higher. For example, Venter, et al.^68^ found the ratio between the Strava data and human counters to range between 1:30 and 1:40 in Oslo, Norway, meaning that for each person using the Strava app there are an additional 30 to 40 people on the same track or road during the same time period. However, as long as Strava users are not using different areas than non-Strava users, this proxy for human activity should reliably represent relative recreational activity in our study area. We believe spatially crowdsourced data on human mobility or activity can open a range of new possibilities for wildlife research and inform management in the future. In cases like ours, with a study area of approximately 43,000 km^2^, crowdsourced data is currently the most feasible, and maybe the only, option for deriving a proxy for human activity across the whole area. The temporal mismatch (in years) between the collection of the lynx data and the Strava data have likely not impacted our results. For this mismatch to have an impact on our results, it has to be a drastic change in recreation patterns between the two periods. We argue that this is unlikely because there was a high correlation among the different years in the yearly Strava data from the period it was collected (Figure S1). However, there are other limitations of the data, e.g. the privacy legislations (minimum 3 unique users on a segment to be reported) impede the use of high resolution temporal Strava data (e.g. activity counts per hour or day) in areas receiving few activity events per temporal unit.

### Conclusions

This study gives an example of the added-value from the application of crowdsourced human mobility data for ecological studies. Our results suggest that lynx reduce their direct interaction with pedestrian recreationists through local-scale avoidance and temporal adjustments in habitat use. The consequences of recreation for lynx in Norway are therefore likely minor as the impact of recreation appears to be spatially restricted to the immediate surroundings of linear features in which the recreation occurs. We believe the levels of recreation in our study area are not high enough to impede lynx from sharing the landscape with humans. Instead, spatial avoidance at local-scales and temporal adjustments in habitat use may facilitate coexistence between humans and large carnivores.

## Supplementary Information

Supplementary Information.

## Data Availability

Data used in this analysis are available from the corresponding author upon request.
