# Supplementary material for: Smartphone app reveals that lynx avoid human recreationists on local scale, but not home range scale
Source: Sci Rep. 2022 Mar 21;12:4787. doi: 10.1038/s41598-022-08468-7 (PMC8938439; doi:10.1038/s41598-022-08468-7)
Supplement: Supplementary file 1 — Supplementary Information. [file 41598_2022_8468_MOESM1_ESM.docx]

**Supplementary material**

**Supplementary material S1 – Description of strava data**

When the Strava app is in use (e.g., during a running or cycling trip) it records the user’s GPS-tracks during their activity (hereafter referred to as an activity event) and provides the user with activity results such as distance, speed, and a route summary for the given activity event. Activity events can also be uploaded to Strava`s webpage after the activity have been recorded by for instance a smartwatch. An activity type (e.g., pedestrian or cyclist) is assigned to each activity event. Every activity event is stored by Strava and aggregated forms of the data can be accessed from Strava Metro. To maintain anonymity and conform with privacy regulations, access is limited to data processed by Strava after removal of personal identifiers and aggregating the data. The processing includes linking individual activity events to nearby linear features (paths, roads etc) in OpenStreetMap (OSM, [www.openstreetmap.org](http://www.openstreetmap.org)). Hence, in the absence of OSM linear features close to the entire activity event (or parts of it), the activity event (or parts of it) is not included in the aggregated version of the dataset (see Figure 2). A linear feature is defined by lines, or a set of lines, connecting two intersections (where two or more linear features intersect), or the start- or endpoint of a line (e.g., the start of path from a parking place to the first path intersection). For each linear feature the number of activity events and the number of unique users for the given activity type are grouped by activity type and counted. For illustration, if five unique users walk or run on the same linear feature five times during a year this will be counted as 25 activity events and 5 users for that linear feature. The timespan for which the number of activity events and number of unique Strava users are counted range from hourly to yearly. Furthermore, to protect user privacy, only linear features with more than 3 unique users are reported and all counts are rounded up to the nearest multiple of 5.

We chose to use Strava data on a yearly temporal scale in our analyses to avoid losing too much of the spatial resolution, i.e., to maximise the spatial cover of the Strava data (the time period to record at least 3 unique users). Furthermore, we decided to use the total number of pedestrian activity events, which excludes biking and the main winter activity of cross-country skiing. We also explored in which land cover classes and what kind of linear features the Strava activities were located (see Figure S2). We used yearly Strava data from 2016 to 2019 and assumed that the spatial patterns of recreation had not changed during the time the lynx had operational collars (2008-2014) and the time the Strava data was collected. In 2020, 9.5 million activity events labelled as pedestrians (running, hiking, or walking) were collected by close to 280 000 unique users in Norway (approximately 5 % of the Norwegian population). The userbase of Strava in Norway has increased since the service commenced. To determine whether patterns of activity events had changed drastically across years we inspected the correlations between the years. The correlation was high (0.97-0.99) for all the combinations of years (see Figure S1), suggesting no conspicuous change in spatial patterns in activity over years.


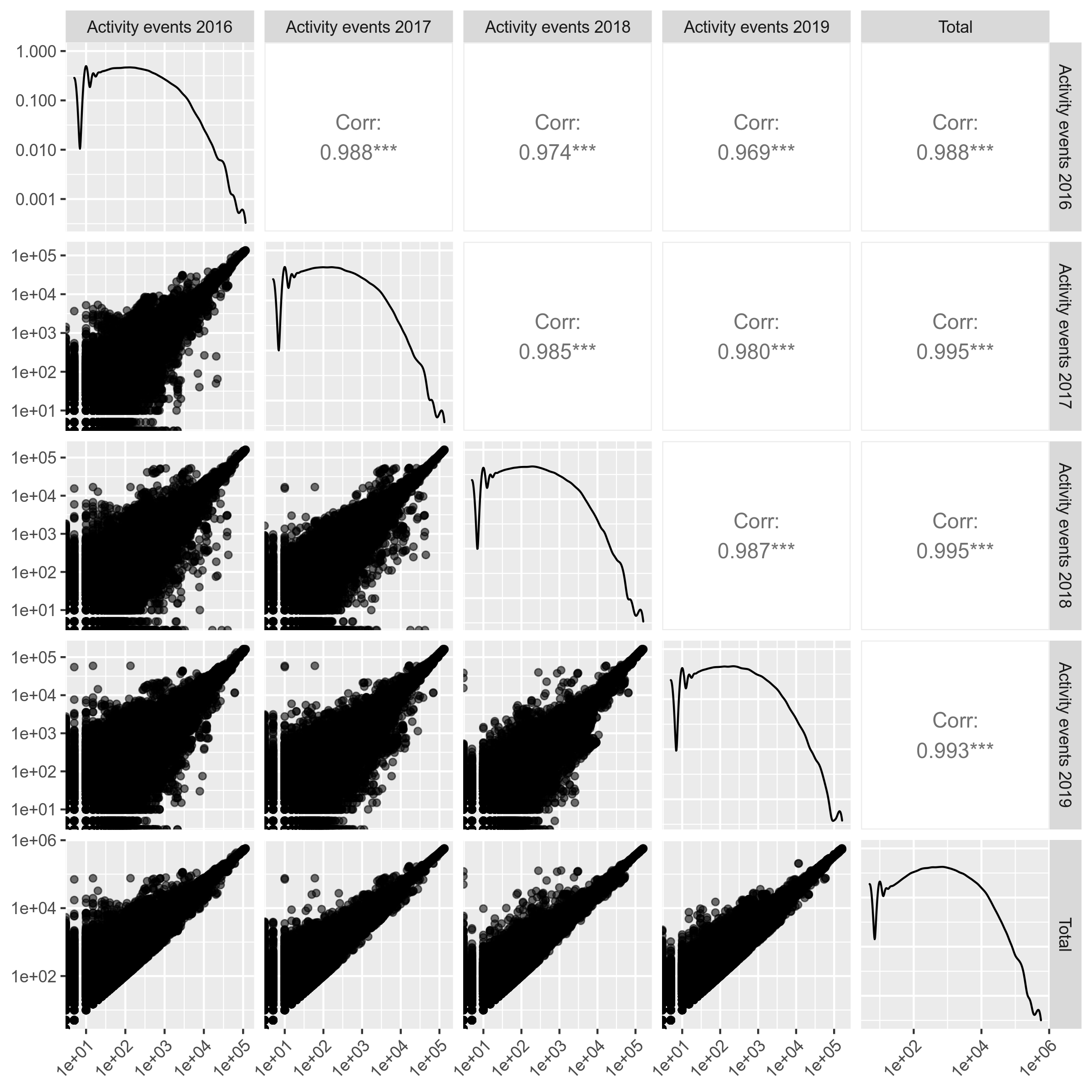


**Figure S1.** Correlation between the number of Strava activity events on linear features in the OpenStreetMap between years. The diagonal panels (from left top to right bottom) are the density distribution for each year and all years combined (“Total”). Values in the panels above the diagonal indicate the correlation between the different combinations of years. In the panels below the diagonal each dot represents a linear feature in the OpenStreetMap. The x-axis in all plots shows the number of activity events. The y-axis for the density distributions show the probability density function, while the y-axis for the panels below the diagonal show the number of activity events. For all panels both axes are given on a log10 scale.


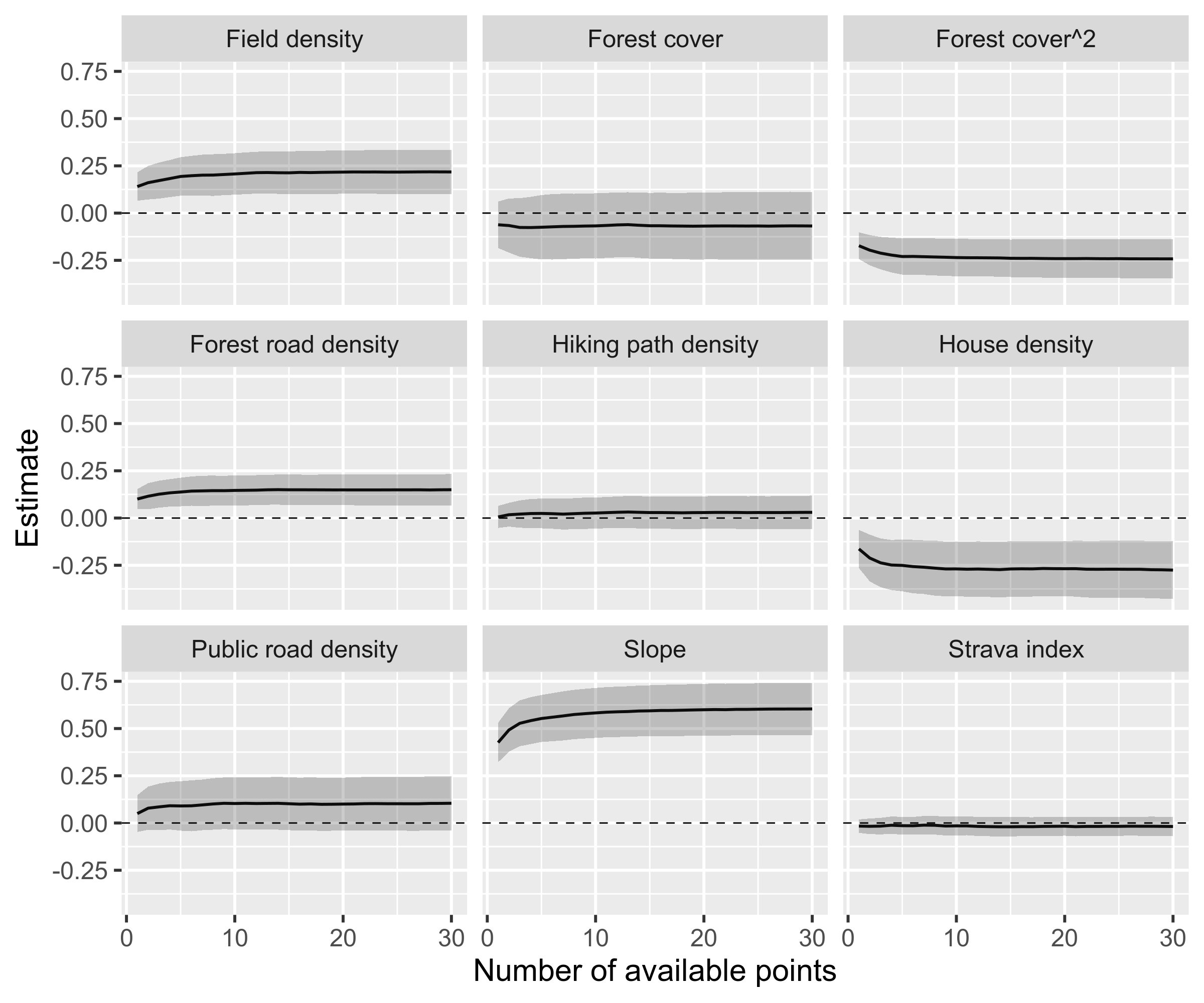


**Figure S2**. Sensitivity analysis for habitat selection at the home range scale. We fitted the model with 1 to 30 available locations. The shaded area is the 95 % confidence interval for the coefficient estimate for the given number of available locations.


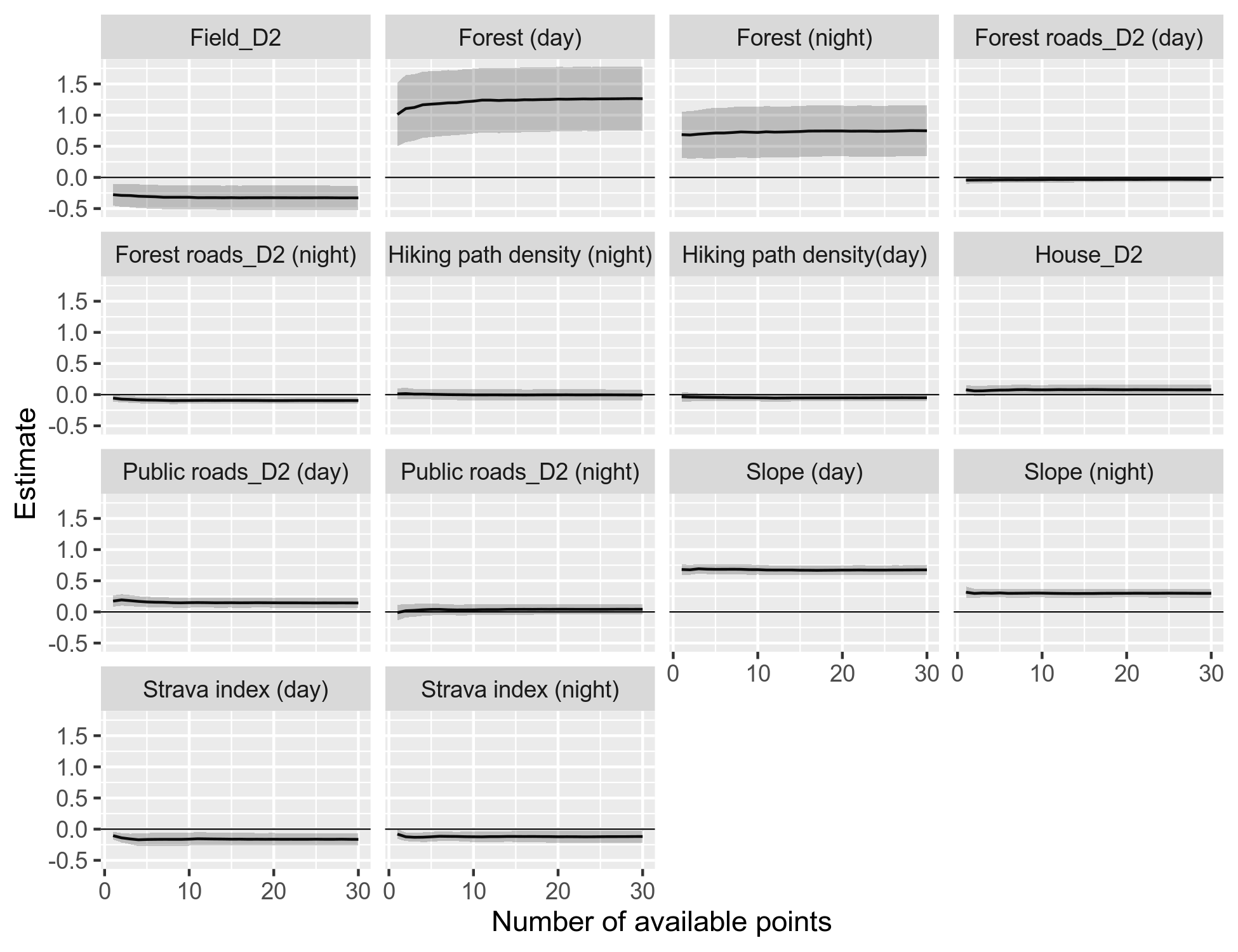


**Figure S3**. Sensitivity analysis for habitat selection at the local scale. We fitted the model with 1 to 30 available locations. The shaded area is the 95 % confidence interval for the coefficient estimate for the given number of available locations.

**
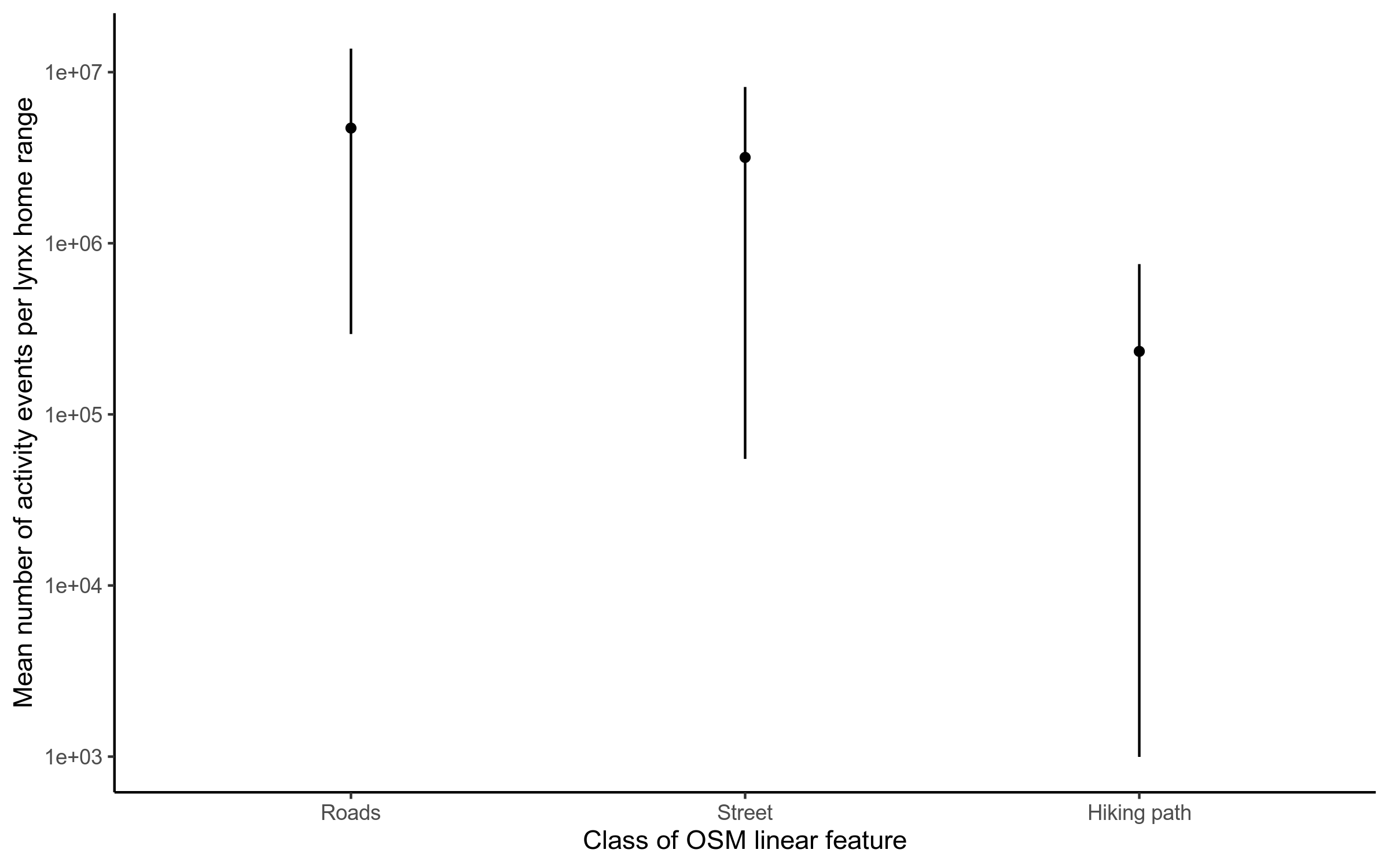
**

**Figure S4.** Mean number of pedestrian activity events inside the lynx home ranges associated with three different classes of linear features in OpenStreetMap (OSM). The black dot is the mean for all home ranges and the lines represent the 10 % and 90 % percentiles. The OSM classes were derived according to the list below. The class “roads” represent all types of roads where cars are able to drive, “hiking path” represent paths and trails that are typically used during hiking (mainly outside urban areas), and “street” represents linear features that are built for pedestrian or cycling activity in urban settings (e.g. sidewalks). The y-axis is shown on log10-scale.

*Roads* contains the following OSM values: Highway.motorway, Highway.motorway_link, Highway.trunk, Highway.trunk_link, Highway.primary, Highway.primary_link, Highway.secondary, Highway.secondary_link, Highway.tertiary, Highway.residential, Highway.unclassified and Highway.service.

*Street* contains the following OSM values: Highway.pedestrian, Highway.living_street, Highway.cycleway, Highway.footway and Highway.steps

*Hiking path* contains the following OSM values: Highway.track and Highway.path.

**Table S1**. Summary of the step lengths and step duration for the individual lynx used in the analysis.

| Individual | Mean step length (m) | Median step length (m) | Standard deviation step length | Mean step duration in hours | Median step duration in hours |
| --- | --- | --- | --- | --- | --- |
| F218 | 727 | 228 | 1165 | 6,4 | 4 |
| F228 | 1058 | 682 | 1272 | 8,4 | 6 |
| F229 | 1246 | 565 | 1898 | 7,3 | 7 |
| F237 | 1816 | 671 | 2797 | 7,1 | 4 |
| F264 | 446 | 111 | 858 | 4,3 | 2 |
| F293 | 839 | 358 | 1170 | 12,8 | 8 |
| F305 | 825 | 365 | 1252 | 7 | 6 |
| F306 | 1615 | 736 | 2215 | 7,3 | 6 |
| M250 | 1139 | 357 | 2208 | 4,8 | 1 |
| M251 | 1055 | 240 | 1895 | 6,8 | 2 |
| M255 | 2029 | 724 | 3431 | 7,8 | 2 |
| M256 | 1233 | 519 | 2102 | 5,2 | 1 |
| M263 | 1949 | 452 | 4603 | 6,2 | 1 |
| M271 | 1311 | 300 | 3209 | 5,1 | 1 |
| M272 | 1210 | 424 | 2828 | 5,1 | 1 |
| M273 | 1221 | 282 | 3088 | 5,2 | 1 |
| M275 | 1343 | 270 | 3062 | 5 | 1 |
| M294 | 1545 | 528 | 3501 | 5,8 | 1 |
| M314 | 1605 | 758 | 2039 | 6,3 | 6 |
| M323 | 1138 | 572 | 1696 | 5,4 | 6 |

**Table S2**. Covariates used for home range-scale habitat selection. The used and available values reported are prior to standardization.

| Covariate | Description | Used values  (mean and range) | Available values  (mean and range) |
| --- | --- | --- | --- |
| Slope_1000_ | Mean of the slope_50_ (see also Table S3) in 1 km radius from focal cell. | 12.4  (0.74 – 28.7) | 10.8  (0 – 35.4) |
| Forest cover | Proportion grid cells with forest in 1 km radius. | 82 %  (8.4 – 100) | 79  (0 – 100) |
| Field density | Percent grid cells with fields in 1 km radius. | 9.9 %  (0 – 86) | 9.3 %  (0 – 98) |
| Forest road density | Sum of forest road length in 1 km radius (km / 3.14 km^2^). | 4.37  (0 – 24.2) | 4.10  (0 – 29.6) |
| Public road density | Sum of forest road length in 1 km radius (km). | 1.77  (0 – 23.8) | 1.68  (0 – 45.2) |
| Hiking path density_1000_ | Sum hiking path length per grid cells in 1 km radius (km). | 2.11  (0 – 58.6) | 2.10  (0 – 62.6) |
| Strava-index_1000_ | Mean number of Strava activities in 1 km radius. | 22.1  (0 – 4.40*10^3^) | 27.0  (0 – 6.24*10^3^) |

**Table S3**. Covariates used for the local-scale habitat selection. The used and available values reported are prior to any transformations.

| Covariate | Description | Day | | Night | |
| --- | --- | --- | --- | --- | --- |
|  |  | Used values (mean and range) | Available values (mean and range) | Used values (mean and range) | Available values (mean and range) |
| Slope_50_ | Calculated as defined by Horn (1981). | 18.1  (0.03 – 57.6) | 12.8  (0 – 68.4) | 14.3  (0 – 57.4) | 12.3  (0 – 68) |
| Forest | Categorical raster where 1 indicates forest. | 0.94  (0 – 1) | 0.85  (0 – 1) | 0.87  (0 – 1) | 0.83  (0 – 1) |
| Distance to field | Euclidean distance to closest fields (km). | 0.70  (0 – 5.81) | 0.74  (0 – 6.10) | 0.67  (0 – 5.7) | 0.72  (0 – 6.34) |
| Distance to forest road | Euclidean distance to closest forest road (km). | 0.61  (0 – 12.8) | 0.63  (0 – 13.4) | 0.67  (0 – 12.1) | 0.69  (0 – 13.0) |
| Distance to public road | Euclidean distance to closest public road (km). | 0.98  (0 – 8.21) | 1.02  (0 – 9.30) | 0.96  (0 – 7.48) | 1.01  (0 – 7.62) |
| Distance to house | Euclidean distance to closest house (km). | 0.85  (0 – 7.15) | 0.89  (0 – 8.31) | 0.84  (0 – 6.11) | 0.89  (0 – 7.34) |
| Density of hiking paths_50_ | Sum hiking path length inside the focal grid cell and its fours closest neighbours (m). | 6.33  (0 – 361) | 8.32  (0 – 626) | 8.52  (0 – 478) | 9.13  (0 – 536) |
| Strava index_50_ | Mean of Strava activities inside the focal grid cell and its fours closest neighbours. | 9.13  (0 – 4.77*10^3^) | 21.5  (0 – 2.02*10^4^) | 14.8  (0 – 4.62*10^3^) | 28.4  (0 – 2.23*10^4^) |

**Table S4**. Model selection for the home range-scale and local-scale habitat selection.

| Model | ∆AIC | |
| --- | --- | --- |
|  | Home range-scale | Local-scale |
| Core | 2.47 | 521 |
| Path | **0** | 503 |
| Strava | 4.46 | 468 |
| Full | 0.08 | 460 |
| Core_ night |  | 64.2 |
| Path_night |  | 43.3 |
| Strava_night |  | 9.17 |
| Full_night |  | **0** |

References
Horn, B. K. 1981. Hill shading and the reflectance map. Proceedings of the IEEE **69**:14-47.
